# Anakinra and hepatotoxicity in pediatric rheumatology: a case series

**DOI:** 10.1186/s12969-023-00891-y

**Published:** 2023-10-06

**Authors:** Frederico Rajão Martins, André Costa Azevedo, Sara Ganhão, Francisca Aguiar, Mariana Rodrigues, Iva Brito

**Affiliations:** 1 Rheumatology Department, University Hospital Centre Algarve, Faro, Portugal; 2 Pediatrics Department, Local Health Unit Alto Minho, Viana do Castelo, Portugal; 3 Pediatric and Young Adult Rheumatology Unit, University Hospital Centre São João, Porto, Portugal; 4 Faculty of Medicine, University of Porto, Porto, Portugal

**Keywords:** Anakinra, Hepatotoxicity, Systemic juvenile idiopathic arthritis, Kawasaki disease

## Abstract

**Background:**

Anakinra is a recombinant interleukin-1 (IL-1) receptor antagonist used in systemic juvenile idiopathic arthritis (sJIA), refractory Kawasaki disease (KD) and cryopyrin-associated autoinflammatory syndrome (CAPS). Anakinra associated hepatotoxicity, while rare, has been described in several cases in daily practice. ​In this case series the authors describe three pediatric patients with this side effect in the setting of severe macrophage activation syndrome (MAS) in KD and sJIA.

**Case presentation:**

The first patient was a 12-year-old boy who presented with fever, maculo-papular exanthema and polyarthralgia. Tonsillitis, distal limb induration and tender cervical lymph nodes were observed. Erythrocyte-sedimentation rate (ESR), C-reactive protein (CRP), ferritin (11,975 ng/mL), D-dimers (5,98 mg/L FEU) and soluble CD25 (3645 pg/mL) levels were elevated. Exclusion of sepsis / toxic shock syndrome warranted introduction of IV methylprednisolone and immunoglobulin (IG IV), with partial response. A MAS secondary to KD was assumed, and anakinra 2 mg/kg/day was introduced. Twenty days later he developed new-onset nausea and severe cyto-cholestasis, normalizing after 2 months of drug discontinuation. Posterior onset of polyarthritis and evanescent lead to a final diagnosis of sJIA. The second patient was a 2-year-old boy with a 10-day history of fevers, generalized rash, hepatosplenomegaly and strawberry tongue. Leucocytosis with neutrophilia and elevated CRP were observed. Initial treatment with IVIG in the setting of incomplete KD was ineffective. Mild anaemia, leukopenia and very high serum ferritin (maximum 26,128 ng/mL) ensued. Presumptive sJIA associated MAS was treated with IV methylprednisolone and anakinra 2 mg/kg/day, with prompt response. Four weeks later transaminitis was detected, and temporary anakinra suspension led to normalisation of laboratorial values. The third case related to a 4-year-old boy presenting with fever, maculopapular rash and cervical lymphadenopathy. CRP and ESR were elevated, and KD was diagnosed. IVIG and methylprednisolone were initiated with clinical worsening, warranting for anakinra introduction at 2 mg/kg/day. After three weeks, liver enzymes progressively elevated, resolving on 2 weeks of anakinra discontinuation.

**Conclusions:**

To the best of our knowledge, this is the first case series describing anakinra associated hepatotoxicity in pediatric patients with rheumatic diseases other than sJIA, bringing additional insight to therapeutic monitoring in patients undergoing this treatment.

**Supplementary Information:**

The online version contains supplementary material available at 10.1186/s12969-023-00891-y.

## Background

Anakinra is a recombinant interleukin-1 (IL-1) receptor antagonist, exerting anti-inflammatory and immunomodulatory actions. There are numerous clinical applications in pediatric rheumatology, including systemic juvenile idiopathic arthritis (sJIA), refractory Kawasaki disease (KD) and cryopyrin-associated autoinflammatory syndrome (CAPS). Off-label use in diverse autoinflammatory diseases such as severe refractory macrophage activation syndrome (MAS) and secondary pediatric hemophagocytic syndrome (SHS) are increasingly debated [1].

Hepatotoxicity associated with anakinra, while having been reported in less than 1% of patients in clinical trials [2], has been described in several cases in daily practice. Its onset varies between a few weeks after initiation up to 6 months, with a clinical presentation similar to an acute viral hepatitis – a hepatocellular pattern of enzymatic elevation, high levels of ALT and AST and mild to moderate jaundice. The clinical course of this condition is generally self-limited within 2 to 8 weeks after suspension of this therapy, generally without sequelae [2].

Hepatotoxicity in pediatric rheumatology has been previously reported in patients with MAS secondary to sJIA undergoing anakinra^34^. Alteration of liver enzymes in these patients may have numerous aetiologies, ranging from mild elevations in initial uncontrolled disease, to serious liver injury secondary to MAS or treatment. Other drugs responsible for hepatic injury in these patients (e.g. methylprednisolone) may be confounding factor when assessing for anakinra-induced liver injury [3].

In this case series the authors describe three pediatric patients with anakinra associated hepatotoxicity, in the setting of severe MAS in KD and sJIA.

## Case 1

**Fig. 1:**
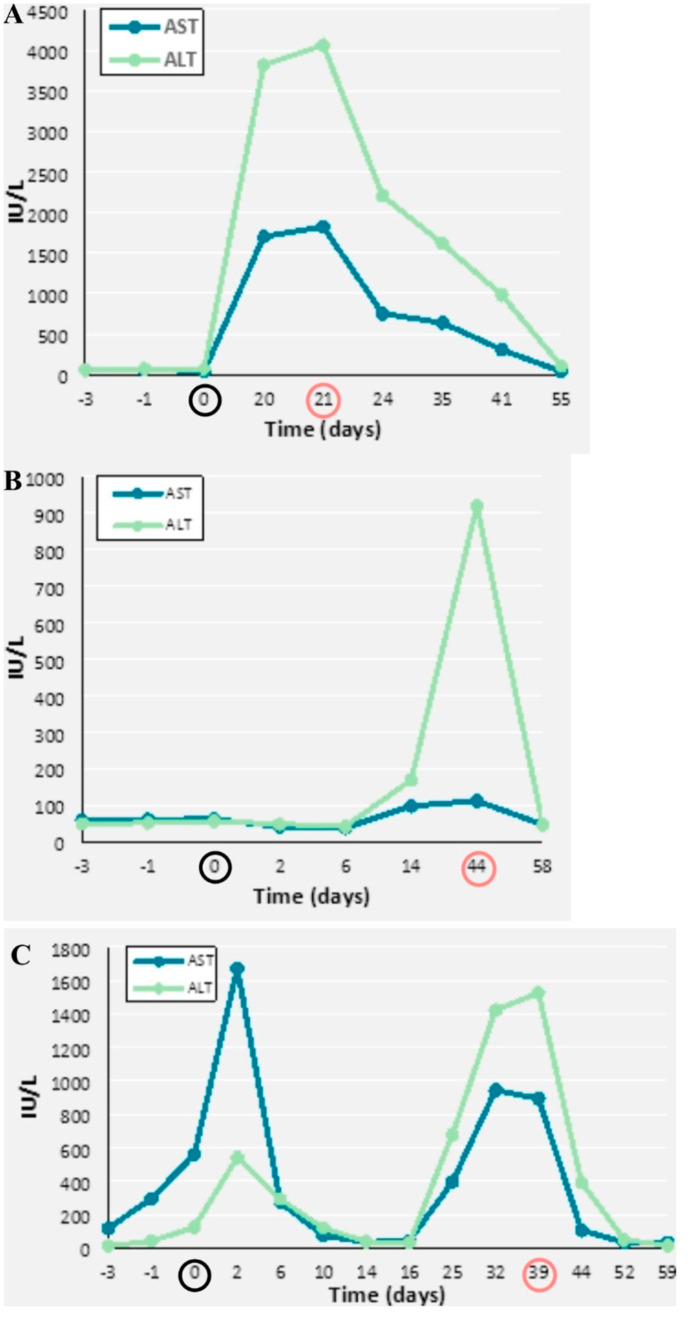
Evolution of liver enzymes after introduction of anakinra in cases 1 **(A)**, 2 **(B)** and 3 **(C)**

A 12-year-old presented in the emergency department with a 48-hour sustained fever associated with unspecific maculo-papular exanthema and polyarthralgia, four weeks after a mild upper airway infection. Tonsillitis, distal limb induration and tender cervical lymph nodes were observed. Laboratorial values showed markedly elevated erythrocyte-sedimentation rate (ESR, 64 mm/hr), C-reactive protein (CRP, 349 mg/L), ferritin (11,975 ng/mL) and D-dimers (5,98 mg/L FEU). Soluble CD25 was elevated (3645 pg/mL). No hepatic dysfunction was noted. Peripheral blood smear, lymphocyte subsets, bone marrow aspirate and biopsy excluded malignancy and showed no haemophagocytosis. Urinalysis and thoracic x-ray were unremarkable. Echocardiogram and electrocardiogram were normal. Due to suspected sepsis / toxic shock syndrome versus KD, the patient received antibiotics, human immunoglobulin (IVIG, 2 g/kg) and aspirin. There was an inadequate response with persistent fevers and macrophagic activation syndrome sedcondary to presumed KD for which she received IV methylprednisolone (30 mg/kg/day) and a second dose of IVIG with partial response, subcutaneous anakinra was then started at a dose of 2 mg/Kg/day, followed by rapid clinical response and normalization of laboratorial values. twenty days later, the patient had mild nausea and severe cyto-cholestasis abruptly installed (Table 1; Fig. 1A). Extensive diagnostic workup was negative: no specific findings on hepatic ultrasound, immunology (anti-nuclear, anti-hepatic, anti-smooth muscle antibodies), or viral serology (Hepatitis A, B and C, SARS-CoV2, Epstein Barr virus, Cytomegalovirus, Parvovirus, Herpes Simplex type 1 and 2). Following drug withdrawal, there was gradual improvement of cyto-cholestasis, after six days of suspension. A diagnosis of anakinra-induced hepatitis was established; liver markers normalized completely after 2 months (Table 1; Fig. 1A). The patient eventually flared with polyarthritis, fevers and an evanescent macular rash and received a final diagnosis of systemic JIA. She is in remission on canakinumab.

**Table 1:** Evolution of cyto-cholestasis markers after anakinra introduction. Day 21, 44 and 39 were the dates of anakinra suspension for cases 1, 2 and 3, respectively. **ALT –** alanine transaminase; **AST –** aspartate transaminase; **GGT –** gamma-glutamyl transferase; **LDH –** lactate dehydrogenase; **RV –** reference values; **TB –** total bilirubin

	*Days after anakinra*	*AST* RV: < 48 U/L	*ALT* RV: < 33 U/L	*GGT* RV: 10–49 U/L	*TB* RV: <1,20 mg/dL	*LDH* RV: < 300 U/L
***CASE 1***	**D0**	40	72	92	-	353
	**D21**	1823	4065	384	2,70	1163
	**D55**	18	47	95	1,37	525
***CASE 2***	**D0**	62	57	29	0,39	389
	**D44**	112	921	129	-	273
	**D58**	47	47	41	0,26	267
***CASE 3***	**D0**	558	124	145	0,30	4116
	**D15**	35	37	205	0,31	554
	**D39**	893	1525	204	0,51	415
	**D59**	33	16	45	0,32	250

## Case 2

A previously healthy 2-year-old boy presented with 10-day history of fevers, maculopapular generalized rash, hepatosplenomegaly and strawberry tongue. Laboratory findings showed marked leukocytosis with neutrophilia and raised c-reactive protein. Due to suspected incomplete KD, he received IVIG (2 g/kg), aspirin and antibiotics.After six days of apyrexia, fever recurred, the evanescent rash became fixed and laboratory findings worsened: mild anemia, decreasing WBC, increasing c-reactive protein and very high serum ferritin (maximum level of 26,128 ng/mL). Echocardiogram was performed in two assessments, with no abnormal findings. A presumptive diagnosis of sJIA with MAS was made and he received IV methylprednisolone (30 mg/kg/day) followed by oral prednisolone and subcutaneous anakinra (2 mg/kg later increased to 3 mg/kg/day) with rapid improvement in clinical and laboratory parameters. Infectious complementary exams were unremarkable, and bone marrow studies excluded lymphoproliferative disease and did not show excess of cells of the monocytic-macrophage system nor apparent increase in hemophagocytic activity. Four weeks later during routine follow-up, elevated hepatic transaminases were detected, which were previously normal. At this point, he was asymptomatic, on anakinra (3 mg/kg/day) and moderate dose of prednisolone (< 1 mg/kg/day). Workup excluded infectious causes of hepatitis. Drug-induced liver injury was suspected and temporary cessation of anakinra lead to a rapid and sustained improvement in liver function tests within 2 weeks (Table 1; Fig. 1B). Anakinra was switched to canakinumab, prednisolone was slowly weaned, and the patient remains asymptomatic. Two years later, he is in remission off-treatment.

## Case 3

A previously healthy 4-year-old boy with a suspected hemophagocytic syndrome secondary to KD was treated with anakinra, which resulted in a marked improvement of the symptoms.

Before starting anakinra, the patient presented a systemic inflammatory condition marked by 2 weeks of intermittent high fevers, maculopapular diffuse fixed skin rash, cheilitis and cervical unilateral lymphadenopathy. Based upon the clinical presentation and laboratory findings of high erythrocyte sedimentation rate and c-reactive protein, KD diagnosis was raised. He was treated with IVIG (2 g/kg), methylprednisolone (3-day IV pulses of 30 mg/kg/day followed by 2 mg/kg/day) and aspirin. Bone marrow aspirate and biopsy were compatible with SHS, excluding malignancy. Despite ongoing treatment, the patient became severely ill, with continuously increasing inflammatory markers, cytopenias, hypofibrinogenemia, hyperferritinemia, hypertriglyceridemia and increasing D-dimersAnakinra was started at 2 mg/kg and the dose progressively increased up to a maximum of 10 mg/kg/day via intravenous route, which finally resulted in improvement of clinical and laboratory parameters. Perineal and extremity desquamation ensued 10 days later. When the patient presented initially, serum aminotransferase levels were elevated, which is in line with SHS/MAS, but after starting anakinra aminotransferase levels decreased progressively until normalization. About three weeks later, serum aminotransferases raised, achieving the maximum level of AST/ALT (aspartate transaminase/alanine transaminase). The patient was completely asymptomatic and blood tests were otherwise normal. Drug-induced liver injury by anakinra was suspected and drug decrease and then cessation led to a rapid improvement in liver injury. Aminotransferases completely normalized within 2 weeks (Table 1; Fig. 1C). Infectious caused were excluded. The patient has been in remission off treatment for 4 years, with no sequelae.

## Discussion

IL-1 plays an important role in inflammation and immunological responses, binding to the IL-1 receptor and activating a wide variety of inflammatory mechanisms. IL-1 receptor is also regulated by a natural IL-1 receptor antagonist (IL-1RA) that binds to IL-1 receptor binding sites and competes with IL-1. Being a recombinant IL-1 receptor antagonist, anakinra binds to the receptor and, therefore, reduces the inflammatory response [4]. Its metabolism in the body is not fully understood. It is known that the main route of elimination is the kidney [5]. Although the non-renal clearance pathway has not been described, as a protein-based therapy it may undergo endogenous proteolysis [5]. Similarly, the mechanism by which anakinra causes liver injury is also unknown. Previous research has related polymorphisms in IL-1 receptor antagonist and IL-1β to antiretroviral hepatoxicity [6]. However, further studies are needed to better understand the direct link between anakinra and liver injury.

Anakinra associated hepatotoxicity is an uncommon side-effect previously observed in the adult rheumatic patient population, especially in individuals with Still’s disease, and several cases of asymptomatic hepatic enzyme elevation have been described [7].

The cases presented are representative of children with rheumatic disease who developed acute hepatitis undergoing anakinra. Clinical presentation was a diagnostic challenge, as diverse infectious and malignant aetiologies had to be excluded. Bone marrow biopsies were not generally suggestive of hemophagocytosis, with the diagnosis of MAS and SHS anchored in clinical and laboratorial data. No apparent triggers were identified in these patients. After exclusion of other aetiologies, anakinra-induced hepatic injury was assumed, resolving after drug suspension. Since they responded to other treatments, no patients were rechallenged with the drug after discontinuation.

Liver enzymes may be elevated during the first stages of sJIA, even in the absence of MAS, although the degree of elevation is generally mild [8, 9]. Moreover, hepatic dysfunction in KD is frequently manifested as transaminitis, despite great individual heterogeneity [10]. Frequently hepatic dysfunction represents the main organ involvement in MAS, presenting as an extreme elevation of liver enzymes and lactate dehydrogenase, besides the more traditional clinical finding of hepatosplenomegaly [11]. Disease-related hepatic dysfunction in these patients is generally accompanied by inflammatory marker elevations, with rapid response to treatment, making anakinra-related hepatotoxicity an exclusion diagnosis [2]. In this case series, all patients had transaminitis when their inflammatory response had improved, thus facilitating differential diagnosis.

Previous studies have reported anakinra associated hepatotoxicity in pediatric rheumatic diseases. Canna S. et al [12] described three cases of anakinra induced acute hepatitis in children treated with anakinra for refractory or severe disease. Only two patients developed suggestive symptoms of abdominal pain and jaundice, with a delay until diagnosis ranging from 44 to 250 days. Hyperbilirubinemia was an important laboratorial feature in two patients and cholestasis was uncommon, with significant AST elevations > 1000 UI/mL in all patients. All laboratorial abnormalities rapidly resolved with therapeutic suspension.

Phadke O. et al [13] described a case series of 3 patients with sJIA/MAS who underwent IL-1 A or IL-6 A treatment, with two patients developing biopsy proven hepatitis less than a month into follow-up of anakinra. There was a significant dissociation between clinical symptoms and inflammatory markers and elevation of liver enzymes, sustaining a drug induced mechanism, as in the cases hereby presented. All situations were reverted with pharmacological discontinuation.

Murray G.M. et al [14] reported a case of a 13-year-old boy with sJIA who was treated with pulsed intravenous MPDN and anakinra 3 mg/kg afterwards with prompt clinical improvement and laboratorial value normalization, with IL-1RA discontinuation after 10 days of treatment. Posteriorly, MAS led to reintroduction of anakinra, with severe elevation of hepatic enzymes 1 month later, with suspension of therapy leading to steadfast cessation of liver injury.

In a recent post authorization safety study using the Pharmachild registry including 306 patients with sJIA, Giancane G et al [15] described adverse events related with anakinra (monotherapy or in association with other glucocorticoids or DMARDs), with description of only 6 cases of increased liver enzymes and hepatitis.

Figure 1 A, B and C show the evolution of liver enzymes after introduction of anakinra in the three cases presented here. The results show an earlier adverse liver injury compared to cases previously described in literature.

Only one patient had hepatic involvement by the rheumatic disease at diagnosis (Case 3), and only one patient had symptoms and objective findings compatible with acute hepatitis (Case 1). A ratio ALT:AST > 1 was observed in all patients, associated with mild to no degree of cholestasis, compatible with a hepatocellular injury pattern, frequently described in acute toxic hepatitis. There was not a clear relationship between dose and degree of liver enzyme elevation, pointing to the fact that anakinra associated hepatotoxicity follows an idiosyncratic pattern in pediatric patients.

Elevation of liver enzymes was observed in the first month of treatment for all patients, as with adult patients who were reported to have developed anakinra-associated hepatotoxicity [7], suggesting a similar pathophysiology for this adverse effect in both subsets of patients.

Liver dysfunction was not more serious in the individual with hepatic involvement by KD at presentation, nor recuperation was longer than in the other cases described. Cessation of anakinra led to rapid resolution of the adverse event in all cases, with an average time until complete resolution of laboratorial abnormalities of 20.3 (± 11.8) days. Data regarding anakinra hepatotoxicity in KD patients is lacking, with previous studies not revealing significant hepatotoxicity, or general side effects for that matter [16].

Liver biopsy may prove useful to ascertain the aetiology of acute liver dysfunction in young patients under anakinra with potential confounding factors, with previous case series describing nonspecific patterns of mixed inflammation without identifiable hemophagocytosis, vascular microthrombi, or cholestatic biliary injury in pediatric sJIA patients [13, 3]. None of the patients in this case series underwent liver biopsy due to causal association between anakinra introduction and hepatic enzyme elevation and swift normalisation of laboratorial values following drug withdrawal, after extensive differential diagnostic consideration and exclusion.

## Conclusion

To the best of our knowledge, this is the first case series describing anakinra associated hepatotoxicity in pediatric patients with rheumatic diseases other than sJIA, bringing additional insight to therapeutic monitoring in patients undergoing this treatment.

This case series highlights a rare side effect of anakinra in the pediatric population, constituting itself a potential confounding factor for an eventual MAS. Larger scale studies for assessment of anakinra-related adverse effects in these diseases are unfeasible owing to their infrequency, thus highlighting the importance of post-authorization registries and case reports.

### Electronic supplementary material

Below is the link to the electronic supplementary material.

Supplementary Material 1

Supplementary Material 2

## Data Availability

All data available are included in this article. Further enquiries can be directed to the corresponding author.

## List of Abbreviations

ALT: Alanine transaminase
AST: Aspartate transaminase
CAPS: Cryopyrin-associated autoinflammatory syndrome
CRP: C-reactive protein
ESR: Erythrocyte sedimentation rate

## References

[1] Maniscalco V, Abu-Rumeileh S, Mastrolia MV (2020). The off-label use of anakinra in pediatric systemic autoinflammatory diseases. Ther Adv Musculoskelet Dis.

[2] Anakinra. *LiverTox Clin Res Inf Drug-Induced Liver Inj*. April 2020. https://www.ncbi.nlm.nih.gov/books/NBK548615/.

[3] Taylor SA, Vittorio JM, Martinez M (2016). Anakinra-Induced Acute Liver failure in an adolescent patient with still’s disease. Pharmacotherapy.

[4] Dayer JM (2003). The pivotal role of interleukin-1 in the clinical manifestations of rheumatoid arthritis. Rheumatology.

[5] Green EA, Metz D, Galinsky R, et al. Anakinra Pilot - a clinical trial to demonstrate safety, feasibility and pharmacokinetics of interleukin 1 receptor antagonist in preterm infants. Front Immunol. 2022;13. 10.3389/FIMMU.2022.1022104. 10.3389/fimmu.2022.1022104 PMC9647081 36389766

[6] Singh HO, Samani D, Nema V, Ghate MV, Gangakhedkar RR. IL-1RN and IL-1 β Polymorphism and ARV-Associated Hepatotoxicity. *Mediators Inflamm*. 2018;2018. 10.1155/2018/4398150. 10.1155/2018/4398150 PMC5911319 29849489

[7] *Kineret. Annex I. Summary of Product Characteristics*; 2021.

[8] Minoia F, Davì S, Horne A (2014). Clinical features, treatment, and outcome of macrophage activation syndrome complicating systemic juvenile idiopathic arthritis: a multinational, multicenter study of 362 patients. Arthritis Rheumatol (Hoboken NJ).

[9] Hiejima E, Komatsu H, Takeda Y (2012). Acute liver failure in young children with systemic-onset juvenile idiopathic arthritis without macrophage activation syndrome: report of two cases. J Paediatr Child Health.

[10] Mammadov G, Liu HH, Chen WX (2020). Hepatic dysfunction secondary to Kawasaki disease: characteristics, etiology and predictive role in coronary artery abnormalities. Clin Exp Med.

[11] Billiau AD, Roskams T, Van Damme-Lombaerts R, Matthys P, Wouters C (2005). Macrophage activation syndrome: characteristic findings on liver biopsy illustrating the key role of activated, IFN-γ-producing lymphocytes and IL-6- and TNF-α-producing macrophages. Blood.

[12] Canna S, Frankovich J, Higgins G (2009). Acute hepatitis in three patients with systemic juvenile idiopathic arthritis taking interleukin-1 receptor antagonist. Pediatr Rheumatol Online J.

[13] Phadke O, Prahalad SR-SK. Reversible hepatotoxicity to IL-1/IL-6 Blockade in Pediatric patients with systemic juvenile idiopathic arthritis and macrophage activation syndrome [abstract]. Arthritis Rheumatol 2020; 72 (suppl 4). https://acrabstracts.org/abstract/reversible-hepatotoxicity-to-il-1-il-6-blockade-in-pediatric-patients-with-systemic-juvenile-idiopathic-arthritis-and-macrophage-activation-syndrome/.

[14] Murray GM, Kheng Ng S, Beasley D, Johansen L, Ramanan AV (2021). Severe hepatotoxicity as a rare side effect of anakinra in a patient with systemic JIA. Rheumatology.

[15] Giancane G, Papa R, Vastert S (2022). Anakinra in patients with systemic juvenile idiopathic arthritis: long-term safety from the Pharmachild Registry. J Rheumatol.

[16] Ferrara G, Giani T, Caparello MC, Farella C, Gamalero L, Cimaz R (2020). Anakinra for Treatment-Resistant Kawasaki Disease: evidence from a literature review. Paediatr Drugs.

