# Supplementary material for: Anakinra and hepatotoxicity in pediatric rheumatology: a case series
Source: Pediatr Rheumatol Online J. 2023 Oct 6;21:112. doi: 10.1186/s12969-023-00891-y (PMC10559407; doi:10.1186/s12969-023-00891-y)
Supplement: Supplementary file 2 — Supplementary Material 2 [file 12969_2023_891_MOESM2_ESM.docx]

Professor Alberto Martini and Professor Charles Spencer,

Editors-in-Chief

Pediatric Rheumatology

Dear Professors,

We wish to submit a case series article entitled “Anakinra and hepatotoxicity in paediatric rheumatology: a case series” for consideration by Pediatric Rheumatology.

We confirm that this work is original and has not been published elsewhere, nor is it currently under consideration for publication elsewhere.

In this paper, we describe a case series of patients with systemic juvenile idiopathic arthritis and Kawasaki disease who developed hepatotoxicity secondary to anakinra used for treatment of macrophage activation syndrome (MAS). Clinical findings were in general nonspecific for acute hepatitis, and laboratorial markers were more reliable in assessing this drug-related toxicity. Hepatic disfunction in MAS was not associated with more severe hepatic disfunction after induction treatment. The adverse events were reversible with drug withdrawal, with no long-term effects on hepatic function or prognosis.

This is significant as anakinra is a frequently administered therapy in children with rheumatic diseases, especially in individuals with auto-inflammatory syndromes. Hepatotoxicity, despite being rarely reported in association with anakinra, is an important side-effect paediatric rheumatologists need to be aware of when prescribing this drug, as it can lead to rapid life-threatening situations.

Given these facts, we believe that the findings presented in our paper will appeal to the clinicians who accompany and subscribe Pediatric Rheumatology. Our findings are of importance in clinical practice, highlighting the need of greater surveillance of hepatic biochemistry, clinical symptoms and physical findings associated with hepatoxicity when using anakinra in young patients.

In case of acceptance of this manuscript for publication, I would kindly ask for the two first authors to be considered and signalled as equally contributing authors.

We have no conflicts of interest to disclose.

Please address all the correspondence concerning this manuscript to me as fredericorajao@gmail.com.

Thank you for your consideration of this manuscript.

Sincerely,

Dr. Frederico Rajão Martins, MD

Doctor, Department of Rheumatology

Centro Hospitalar Universitário do Algarve
